# Evaluation of a Novel HA/ZrO_2_-Based Porous Bioceramic Artificial Vertebral Body Combined with a rhBMP-2/Chitosan Slow-Release Hydrogel

**DOI:** 10.1371/journal.pone.0157698

**Published:** 2016-07-11

**Authors:** Yihui Shi, Renfu Quan, Shangju Xie, Qiang Li, Guoping Cao, Wei Zhuang, Liang Zhang, Rongxue Shao, Disheng Yang

**Affiliations:** 1 Zhejiang Chinese Medicine University, Hangzhou, China; 2 Department of Orthopedics, the Affiliated JiangNan Hospital of Zhejiang Chinese Medical University, Hangzhou, China; 3 Guangxing Hospital Affiliated to Zhejiang Chinese Medical University, Hangzhou, China; 4 Department of Orthopedics, the Second Affiliated Hospital, Medical College of Zhejiang University, Hangzhou, China; University of Oulu, FINLAND

## Abstract

A new HA/ZrO_2_-based porous bioceramic artificial vertebral body (AVB), carried a recombinant human bone morphogenetic protein-2 (rhBMP-2)/chitosan slow-release hydrogel was prepared to repair vertebral bone defect in beagles. An ionic cross-linking was used to prepare the chitosan hydrogel (CS gel) as the rhBMP-2 slow-release carrier. The vertebral body defects were implanted with the rhBMP-2-loaded AVB in group A, or a non-drug-loaded AVB in group B, or autologous iliac in group C. The encapsulation rate of rhBMP-2 in rhBMP-2-loaded CS gel was 91.88±1.53%, with a drug load of 39.84±2.34 ng/mg. At 6, 12, 24 weeks postoperatively, radiography showed that the bone calluses gradually increased with time in group A, where the artificial vertebral body had completely fused with host-bone at 24 weeks after surgery. In group C, an apparent bone remodeling was occurred in the early stages, and the graft-bone and host-bone had also fused completely at 24 weeks postoperatively. In group B, fusion occurred less than in groups A and C. At 24 weeks after surgery, micro-computed tomography (Micro-CT) revealed that the volume of newly-formed bone in group A was significantly more than in group B (p<0.05). At 24 weeks after surgery, ultra-compressive strengths of the operated segments were 14.03±1.66 MPa in group A, 8.62±1.24 MPa in group B, and 13.78±1.43 MPa in group C. Groups A and C were both significantly higher than group B (p < 0.05). At 24 weeks postoperatively, the hard tissue sections showed that the AVB of group A had tightly fused with host bone, and that pores of the AVB had been filled with abundant nearly mature bone, and that the new bone structured similarly to a trabecular framework, which was similar to that in group C. In contrast, implant fusion of the AVB in group B was not as apparent as group A. In conclusion, the novel HA/ZrO_2_-based porous bioceramic AVB carried the rhBMP-2-loaded CS gel can promote the repair of bony defect, and induce bone tissue to grow into the pores, which may replace iliac bone grafts as commonly applied in clinical practice.

## 1. Introduction

In current clinical practice, artificial bone grafting is commonly used to repair large bone defects. The key factor for successful grafting is the ability of new bone tissues to grow into the grafts postoperatively [1]. Specifically, sufficient new bone must form at the graft site and a strong connection must be formed between the graft-bone and the host-bone tissue, while most of current bone grafts act as bone substitutes. The repair of large bone-defect is complicated, and need longer fusion time with an increasing risk of infection and implant collapse. Therefore, improving osteogenesis and fusion rates of artificial bone grafts will certainly promote the success of bone graft surgeries [2–5]. The research of artificial bone material to achieve these improvements has become a popular trend in the field of bone tissue engineering. In this study, the Zirconia (ZrO_2_) was applied as an enhancement substance for Hydroxyapatite (HA) to fabricate a HA/ZrO_2_-based porous bioceramic artificial vertebral body (AVB). The ZrO_2_-HA containing AVB had superior bone conduction activity, with excellent mechanical properties and plenty new bone growing into the designed pores would be desired as an ideal bone scaffold.

In addition, it is commonly accepted that rhBMP-2 mediates bone induction. Almost all bone morphogenetic proteins (BMPs) in mature osteogenic cells can stimulate alkaline phosphatase; however, rhBMP-2 is the only one that induces all osteogenic cells to differentiate in both pluripotent hematopoietic stem cells and mesenchymal stem cells. In July 2002, rhBMP-2 was approved by the Taiwan Food and Drug Administration (TFDA) for use in anterior lumbar fusion. However, the side effects have been revealed occasionally with clinical application of rhBMP-2 over the last 10 years [6–7]. Firstly, purified rhBMP-2 has short half-lives and diffused easily after implantation, which is dissolved readily following contact with bodily fluids and enzymes [8]. Secondly, single high-dose rhBMP-2 has many side effects, such as early osteolysis around the bone graft, postoperative spinal edema, and spinal cord heterotopic ossification [9–11]. Therefore, carrier is needed to deliver rhBMP-2 and allow for its slow and local release under a stable concentration to achieve a therapeutic efficacy [12, 13]. Chitosan is often used as a slow-release carrier because it is a natural material, with nontoxic, biocompatible and biodegradable characteristics [14]. Additionally, it also has hemostatic and anti-cancer properties [15]. The chitosan has a positive charge and NH_2_ groups, which allows it to interact with negatively-charged polymers and macromolecular proteins. Therefore, it has been widely used in the biopharmaceutical field and for designing slow drug-release systems [16, 17]. In present study, chitosan hydrogel was used as a slow-release carrier for rhBMP-2; and the ZrO_2_ was applied as the reinforcement for HA to prepare a HA/ZrO_2_-based porous bioceramic AVB. Herein, we tested whether it has superior biocompatibility, osteogenic activity and adequate biomechanical strength to promote the repair of bony defect, in hope to establish an experimental basis for clinical treatment of bone defects.

## 2. Materials and Methods

### 2.1 Animals, reagents and equipment

Animal experiment was approved by Institutional Animal Care and Use Committee of Zhejiang Chinese Medical University, and performed by strictly following the Chinese Law on animal experimentation. Fifteen Male beagle dogs (5–6 months old, 10.3–12.5 kg), were provided by the Experimental Animal Center, Zhejiang Chinese Medicine University, China (Number of qualitative qualification SCXK (Su) 2010–0002). The following reagents were used: Chitosan, (molecular weight 190k-220k, degree of deacetylation > 90%; Sigma, USA); sodium tripolyphosphate (STPP; Sigma, USA); recombinant BMP-2 (Sigma, USA); ELISA kit (R&D, USA); lyophilizer (Labconco Co., USA); enzyme-linked immunosorbent assay (ELISA); microplate reader (Thermo Scientific Varioskan Flash); micro-CT (Skyscan Bruker, Belgium); scanning electron microscope (SEM; S-3000N Hitachi, Japan); sputter coater (Hitachi E-1010); material testing system (Instron 5569, USA).

### 2.2 Preparation of rhBMP-2 chitosan hydrogel

#### 2.2.1 Chitosan hydrogel preparation

Chitosan (CS) was dissolved in 0.3% acetic acid to a final concentration of 1.5%. STPP was dissolved in water to a final concentration of 0.5 mg/mL. CS: STPP solution (5:1, v/v) was prepared: CS and STPP were both passed through 0.45 μm and 0.22 μm filters, and STPP was then slowly added to CS (100 mL) using a syringe (gauge 7) at a rate of 30 drops/min under continuous magnetic stirring (350 rpm/min). The solution color gradually changed to blue and was stirred continuously for 1 h after STPP was added up. Finally, 0.5% NaOH was used to adjust the pH to 7.2–7.4. The cross-linked solution was aliquoted into culture plates and then lyophilized. All the preparation procedures were carried out under sterile conditions.

#### 2.2.2 Observation under SEM

The lyophilized CS gel was cut into small pieces (3 mm x 1 mm) and fixed on the material testing system, followed by coating with gold using the sputter coater. SEM was used to observe the 3-D structure of the lyophilized gel and obtain its images.

#### 2.2.3 Determination of drug-loading and the encapsulation rate of the chitosan-rhBMP-2 complex

An unloaded and dried CS gel was prepared, which had swelling property that allowed the CS/rhBMP-2 compound to form when the rhBMP-2 solution was added. The drug load and the corresponding encapsulation rate of the rhBMP-2 were quantified by measuring the residual amount of rhBMP-2 left at the bottom of tubes. Specifically, 40 mg dried CS gel was added into three 2-ml centrifuge tubes; 100 μL of 20 μg/mL rhBMP-2 was added dropwise into each tube, followed by incubation at 37°C in the presence of a magnetic field to prepare gelation; the CS hydrogel was removed when it turned to white color from light yellow. The gel was removed to avoid contact with the centrifuge tube wall; 1 mL of phosphate-buffered saline (PBS) was added into the bottom of each centrifuge tube, followed by vortexing for 5 mins to promote residual rhBMP-2 to be completely dissolved. A high-sensitivity ELISA kit was used to determine the concentration of rhBMP-2 in the solution and the total rhBMP-2 content in the rinse solution.

$$\text{Drug load}\;(\text{ng}/\text{mg})=\frac{\text{rhBMP}2\;\text{added-rhBMP}2\;\text{left in microcentrifuge}}{\text{total CS content}}$$

$$\text{Encapsulation rate}\;(\%)=\frac{\text{rhBMP}2\;\text{added}-\text{rhBMP}2\;\text{left in microcentrifuge}}{\text{rhBMP}2\;\text{added}}\times 100\;\%$$

#### 2.2.4 Determination of the cumulative release rate of rhBMP-2 from the CS-rhBMP-2 compound in vitro

Five dried CS gels (40 mg each) were added to the bottom of 5-mL test tubes, followed by dropwise addition of 100 μL of 20 μg/ml rhBMP-2. The mixture was placed in an incubator at 37°C to initiate the gelation process with the application of a magnetic field. When the RhBMP-2/CS compound turned yellow, 2 mL PBS (pH 7.4) containing 0.1% bovine serum albumin was added into each tube, followed by further incubation at 37°C. Supernatants (100 μL) from each tube were tested for rhBMP-2’s release at different time points (days 0.5, 1, 2, 3, and then every three days) using a high sensitivity ELISA kit. The same amount of PBS containing 0.1% bovine serum albumin was added to maintain the final volume when the supernatant sample was taken each time. The amounts of rhBMP-2 were calculated and a cumulative releasing-curve was created.

$$\text{rhBMP}2\;\text{releasing rate}=\frac{\text{rhBMP}2\;\text{concentration in supernatant}\times 2\;\text{mL}}{2\;\mu g}\times 100\%$$

### 2.3 Osteogenic activity of rhBMP-2 in the novel HA/ZrO_2_ porous bioceramic AVB

#### 2.3.1. Preparation and confirmation on surface characteristics of the HA/ZrO_2_ porous bioceramic AVB

Organic polyurethane foam was cut into half-cylinder (24 cm in height and 9 cm in diameter) and then soaked in 15 wt% NaOH at 60°C for 3.5 h. The soaked half-cylinders were rinsed with water three times and dried. The half-cylinders were further processed using the surface-active agent, and finally soaked in 5% polymercaptans curing agent (PCA) for 24 h before drying. The obtained foam half-cylinders were coated with ZrO_2_ powder and then subjected to step-wise sintering as follows: drying was performed by increasing from room temperature to 100°C at a rate of 2°C/min to evaporate the residual water in the initial mold. Then, the temperature was increased from 100 to 200°C at a rate of 1°C/min, followed by an increase from 200 to 500°C at a rate of 1°C/2 min. The temperature was maintained at 500°C for 1 h; a further increase in temperature was performed from 500 to 750°C at a rate of 2°C/3 min., and maintained at 750°C for 1 h. The temperature was then increased from 750 to 1,200°C at a rate of 2°C/min. The high-temperature sintering stage was performed by increasing the temperature to 1,550°C at a rate of 3°C/min, which was maintained for 3 h, followed by a cooling stage. The ZrO_2_ AVB (23 cm high and 9 cm in diameter) was consequently prepared.

**Fig 1:**
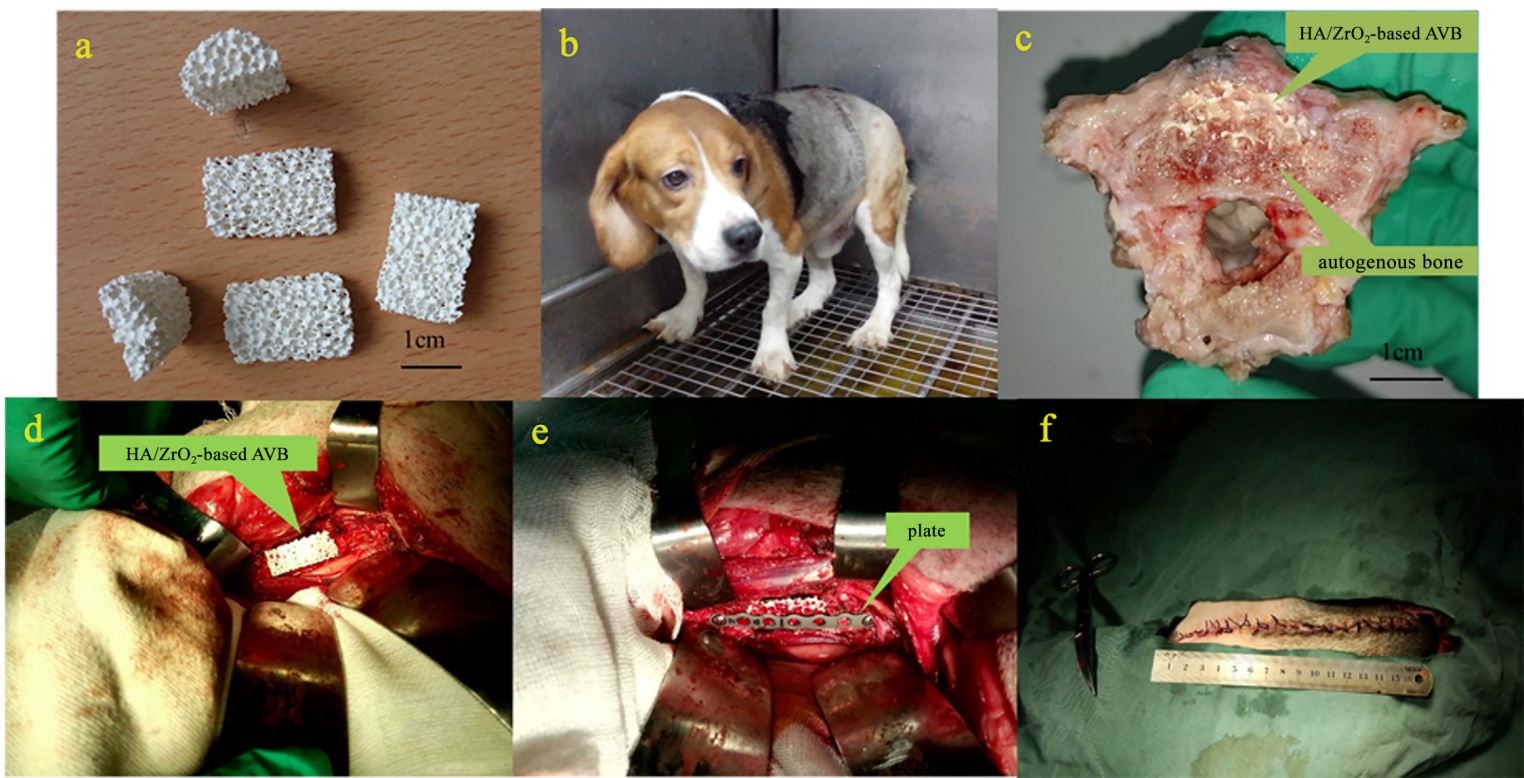
Animal experimental situation. (a) the prepared half-cylinders HA/ZrO_2_ porous bioceramic AVB; (b) the beagle dog could stand independently when recover postoperative; (c) postoperative specimens shown: the implanted AVB had integrated with autogenous bone at week 24; (d) embedded AVB in the vertebrae defect in operation; (e) a 56-mm-long surgical steel plate was used to stabilize the implanted AVB; (f) wound closure at last.

Gradient HA coating was performed in two steps. In step 1, the solution of coating material included 31.1% ZrO_2_ powder, 13.3% HA powder, 1.4% ethyl phosphate, 0.2% ethyl cellulose, and 53% ddH_2_O. Briefly, the HA power was pretreated at 800°C for 2 h, and then mixed with the remaining ingredients at 50°C for 5 h. The sintered ZrO_2_ mold previously was soaked completely into the solution of coating material, and then dried for 5 h at 100°C after the extra coating was removed. The mold was then sintered at 900°C for 5 h and then 1,250°C for 1 h. In step 2, the solution of coating material contained 3.9% ZrO_2_ powder, 35.5% HA powder, 1.4% ethyl phosphate, 0.2% ethyl cellulose, and 58% ddH_2_O. The coating process was repeated as performed in step 1. Finally, the half-cylinders HA/ZrO_2_ porous bioceramic AVB, with dimensions of 24 cm in height and 9 cm in diameter, was prepared (Fig 1).

The surface roughness and internal voids situation of HA/ZrO_2_ porous bioceramic AVB were observed by ultra-depth microscope and SEM. And the D/max-2550 X-Ray diffractometer was applied to analyze the phase composition of HA/ZrO_2_ porous bioceramic AVB.

#### 2.3.2. Preparation of the rhBMP-2/CS AVB

Regarding preparation of the unloaded and dry CS AVB, CS was completely dissolved into 0.3% acidic acid with stirring, and the final concentration was controlled at 1.5 mg/mL. STPP was dissolved into super-purified water to a final concentration of 0.5 mg/mL. The CS: STPP solution (5:1, v/v) was prepared as described in section 1.2.1. The cross-linked solution was aliquoted into a culture plate. The HA/ZrO_2_ porous bioceramics AVBs were soaked into the prepared CS solution and then lyophilized. The lyophilized HA/ZrO_2_ porous bioceramic AVBs carried the dried cross-linked CS in the inner pores. The AVBs were sterilized using ethylene oxide and stored subsequently.

The AVBs and rhBMP-2/CS combination were done during the surgery. Five hundred microliters of 20 μg/mL rhBMP-2 solution were added dropwise to the sterile HA/ZrO_2_ porous bioceramic AVBs containing the dried and unloaded cross-linked CS so that the rhBMP-2 could be absorbed completely. A total of 10 μg of rhBMP was loaded into each AVB.

#### 2.3.3 Animals and surgery

A total of 15 adult beagle dogs were randomly assigned into three groups, which were implanted with rhBMP-2/CS AVB grafts in group A (n = 5) or non-drug-loaded/ CS AVB grafts in group B (n = 5) received, or autologous iliac bone grafts in group C (n = 5). Intravenous anesthesia was performed using 3% pentobarbital at a dose of 1 mL/kg, followed by tracheal intubation, which would provide the sustained oxygen supply during the surgery. The animals were placed in a right lateral position. The surgery began at the front lateral lumbar with an incision of 8–12 cm in length, and the lower back skin and the subcutaneous tissues were then cut open to locate the lumbar transverse process by touching the last rib. The blunt muscle dissection was performed between the psoas muscles at the L4 level to fully free the bilateral transverse arteries and the parallel veins at this level, which were subsequently ligated. The abdominal aorta and the accompany vena cava were separated and protected with saline gauze, so that the L4 vertebral body and half of the superior and inferior vertebral bodies were fully exposed. Two-thirds of the vertebral bodies along the intertransversarii at the coronal plane were cut away. The surgical procedure generated a semi-cylindrical bone defect with dimensions of 9 mm × 18 mm × 23 mm. Fixation of the AVBs was confirmed and then a 56-mm-long surgical steel plate was screwed in to temporarily stabilize the vertebral bodies (Fig 1). Muscles, subcutaneous tissues, and skin tissues were sutured in layers (Fig 1). All bone defect modeling, blood vessel ligation, material placement and steel plate fixation procedures were performed by the same team of staff.

The dogs were housed in the Zhejiang Chinese Medicine University vivarium on meshed floor in a room with a fenced area about 3.8 × 3.8 meters (Fig 1). The room number in which the animals were housed throughout the study period was detailed in the study records. The room in which the dogs were housed is an area within the facility that had filtered air ventilation at the rate of 10–20 air changes per hour). The temperature was maintained at 16–26°C (61–79°F) with a relative humidity of 40–70%. Illumination was fluorescent light for 12-hour light (08:00–20:00) and 12-hour dark. A unique number is assigned to each dog. The room is labeled with cards identifying study number, species/strain, sex, cage number and animal numbers. Dogs had ad libitum access to dog food (Beijing Keaoxieli Feedstuff Co. Ltd.). Water from the Animal Center of Zhejiang Chinese Medicine University in house production is available to dogs ad libitum throughout the study period. Water from the municipal water supply is purified by filter system and meets WHO human drinking water standard.

A unique number was assigned to each dog post-surgery. There was intermittent socialization with other dogs of 20–30 minutes per day. The dogs were provided with positive human interaction daily by animal care staff. The dogs would continue to receive Buprenorphine (minimal at 0.02 mg/kg, i.m.) for 2 additional doses at about 8 hours apart for 3 postoperative days; and again as deemed necessary. If animals show signs or symptoms of more than momentary pain/discomfort and/or distress that cannot be relieved with analgesic drugs will be immediately euthanized. The dogs received intramuscular penicillin (1,600 KU) each day for three consecutive days to prevent infection.

#### 2.3.4 Postoperative general situation and X-ray observations

The standing behavior of each beagle was recorded immediately after surgery. Their activities were measured at 6, 12 and 24 weeks. Briefly, the jumping-frequencies per minute were recorded under the same type of food treats and the same height set. Recordings were made in triplicate and analyzed using paired *t*-test. At 6, 12 and 24 weeks after surgery, the radiography of lateral lumbar was performed under intravenous anesthesia to observe the bone callus growth surrounding the grafted porous bioceramic AVB and the height changes of the replaced vertebral segment. The exposure factors of radiography were 44 kV, 100 mA, 0.6 MS.

#### 2.3.5 Specimen harvest after surgery

There are none the animals became severely ill or died at any time prior to the experimental endpoint. At week 24 postoperatively, dogs were given anesthesia (Zoletil, 3-4mg/kg body weight) first and injected with potassium chloride to cause cardiac arrest. Death was confirmed with stethoscope on ceased heart beat and respiration. The operated lumbar segments were exposed and obtained. The adjacent vertebral bodies and surrounding soft tissues of the lumbar vertebral body were removed. The lumbar vertebras of operated segment with the intact facets were preserved at −20°C. So that five specimens were obtained from each group, one for making hard tissue section, and another four specimens for micro-CT to examine new bone amount and test biomechanical properties.

#### 2.3.6 Micro-CT scans on the volume of newly-formed bone in the AVBs’ area and the 3D reconstruction images

At 24 weeks, the vertebral specimens were subjected to 180° horizontal x-ray scanning, and the region of interest (ROI) covered all of the AVB’s area. The ultra-high signals from the material were filtered using the CT signal selection function, and approximate 200 two-dimensional (2D) images of the newly-formed bone were obtained with a section thickness of 50 μm. The bone volume (BV) of the newly-formed bone was calculated by micro-CT. Five 2D-images (with of total thickness of 250 μm) near the upper surface of the material were used to reconstruct the 3D images of the newly-formed bone with the embedded software.

#### 2.3.7 Biomechanical testing

The UCS of an overall vertebral body fused with the porous bioceramic AVB (group A or B), as well as the autologous bone grafted (group C) was tested and recorded. The structure imitation, loading speed, and the specimens’ fixation methods were maintained constant to improve the test precision. The average surface length and width of each vertebral body was measured using a Vernier caliper for calculating the surface area (S) of the stress interface of each vertebral body. The specimens were mounted and fixed in a biomechanical testing system along a plane parallel to the long axis of the implants. The loading speed of the test machine was 0.5 mm/s, and the load increased from zero until the specimens were destroyed. A CAD automated recorder recorded the maximum compressive load (Fm). The UCS was defined as: *P = Fm/S*, where the *S* referred to the area loaded surface.

#### 2.3.8 Production of Hard tissue section stained with toluidine blue

At 24 weeks after operation, the new-bone formation and fusion were observed using hard tissue toluidine blue staining of each group. Specimens were degreased with acetone dehydration for seven days (replaced liquid twice a day, rate of fluid and sample volume is 10:1), methyl methacrylate-embedded, 7 d in an incubator at room temperature. The above specimens were cut and grinded to 150 μm thickness by Reichert-Jung microtome, and stained by toluidine blue and observed by an upright bright-field light microscope.

## 3. Results

### 3.1 SEM observation of the lyophilized gel

**Fig 2:**
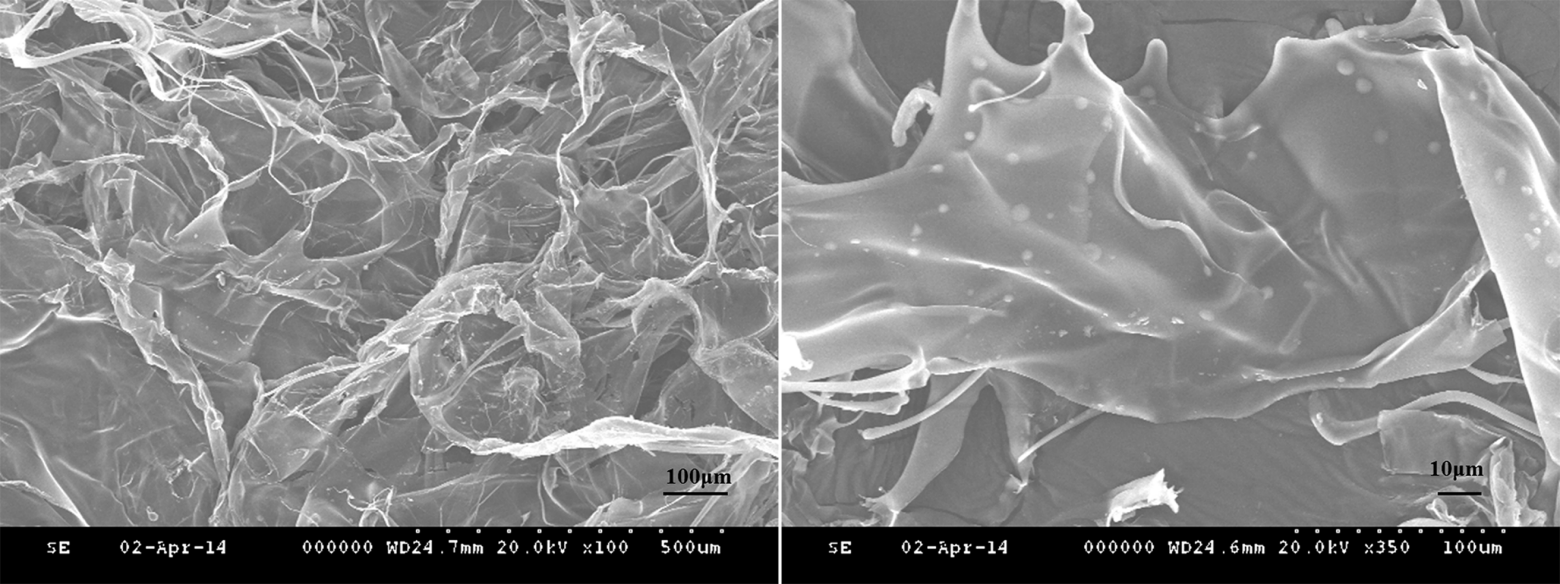
Scanning electron microscopy images of lyophilized hydrogel. **(A)** the lyophilized hydrogel observed as an three-dimensional mesh structure (× 100); **(B)** the round chitosan microspheres with smooth surface were evenly distributed on the film-like surface (× 350)

The lyophilized CS gel had a 3D mesh film structure under SEM. The diameters of 3D pores were between 50–300 μm. The micro-sized CS microspheres with smooth surfaces were evenly distributed on the film surface (Fig 2).

### 3.2 Determination of the drug loading and encapsulation rate of the rhBMP-2/CS compound

The encapsulation efficiency of the rhBMP-2 in CS hydrogel was 91.88 ± 1.53%; and the corresponding loading capacity was 39.84 ± 2.34 ng/mg.

### 3.3 Illustration of the cumulative release rate of the rhBMP-2/CS compound in vitro

**Fig 3:**
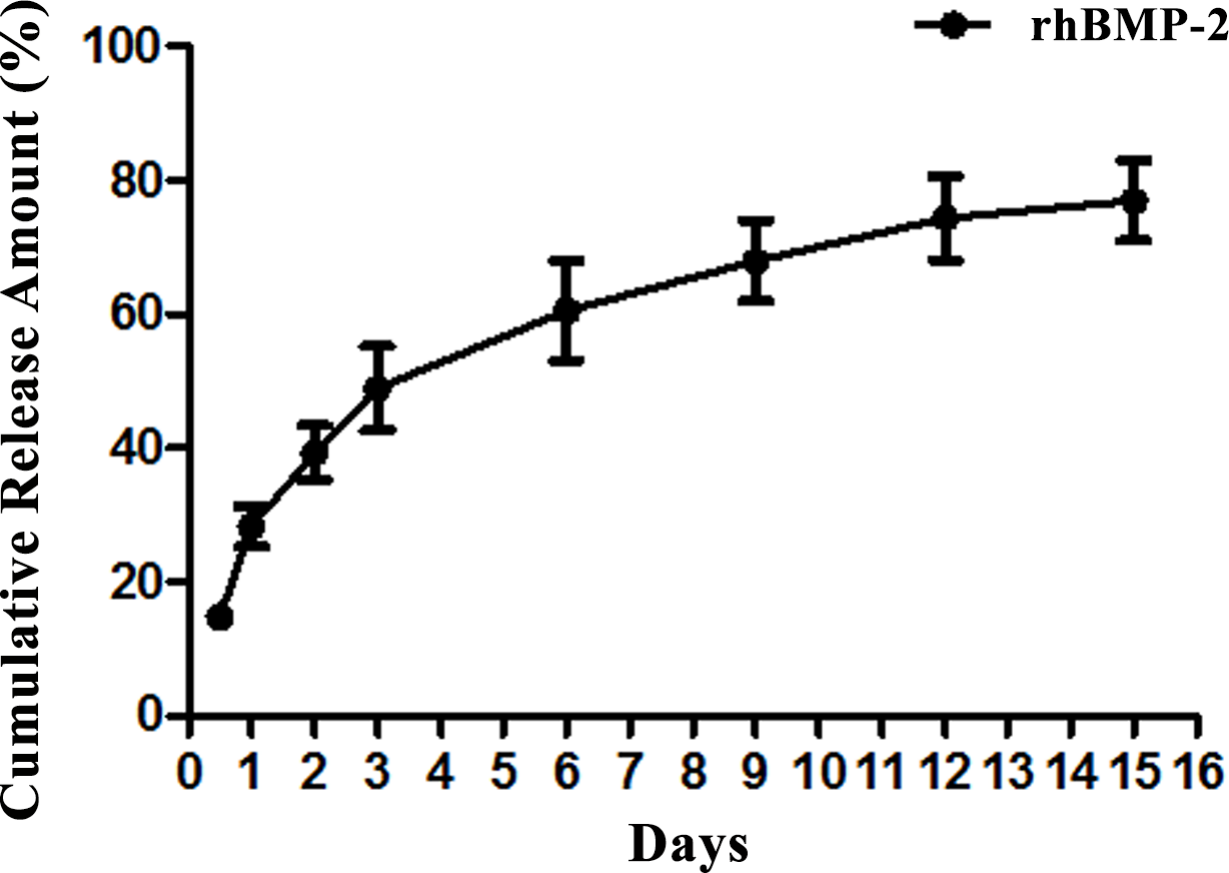
In vitro release curve of the chitosan hydrogel complex with rhBMP-2.

The cumulative release of rhBMP-2 was expressed as the percentage of released rhBMP-2 compared with the loaded total. As shown in Fig 3, the rhBMP-2 was released in different stages. such as the burst-release in the first 3 days with 28.32 ± 3.01% released on day 1, and as high as 48.92 ± 6.27% by day 3; the slower-release from days 3–12 with the cumulative release of 74.40 ± 6.29% at day 12; and, the steady-release between days 12–15 with a cumulative release of 76.97 ± 6.05% at day 15.

### 3.4 Surface characteristics detection of HA/ZrO_2_ porous bioceramic AVB

**Fig 4:**
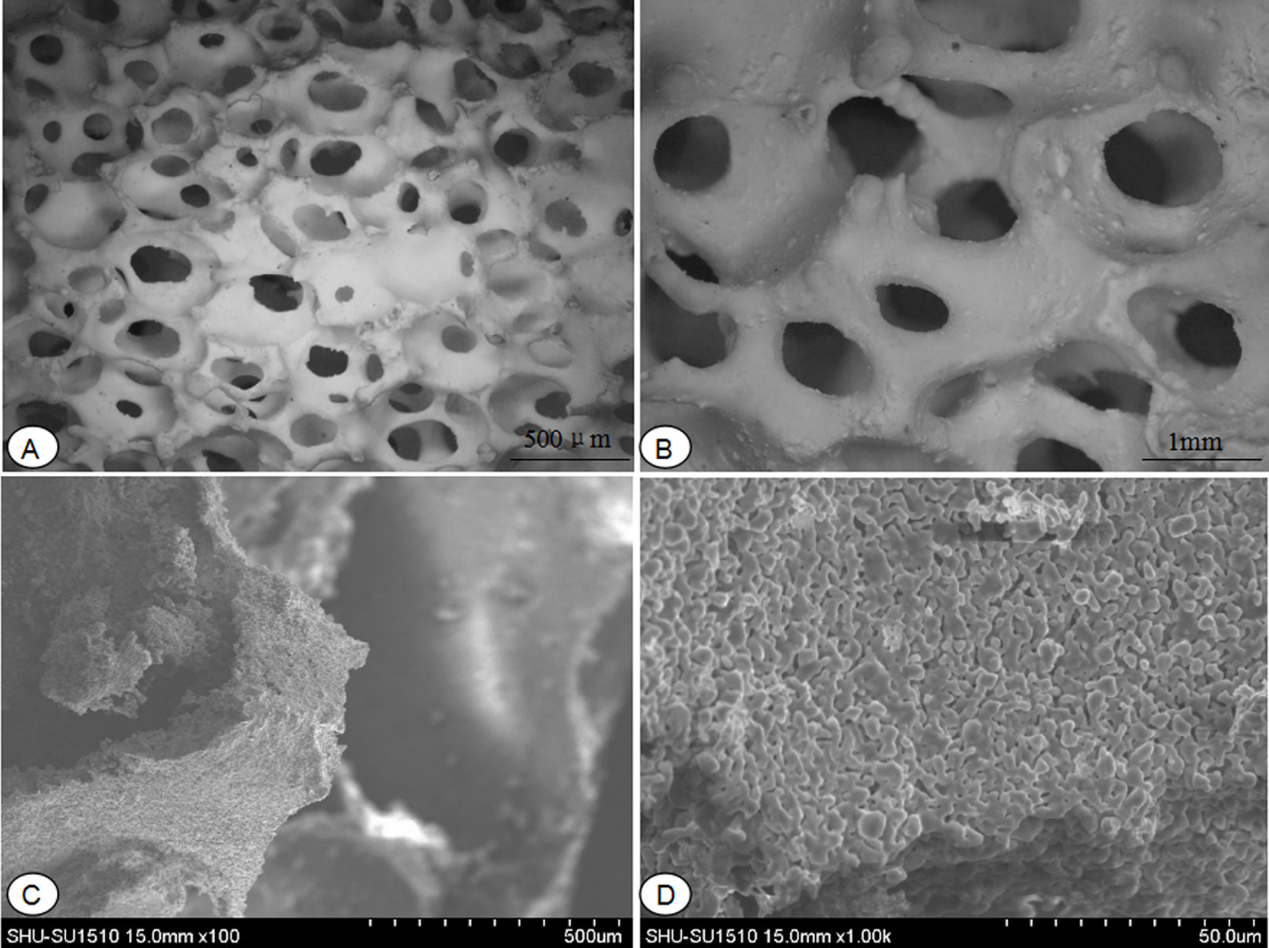
Ultra-depth microscope observation of HA/ZrO_2_ porous bioceramic AVB. **(A)** under a 10 times magnifying glass; **(B)** under a 20 times magnifying glass; **Scanning electron microscopy images of HA/ZrO**_**2**_ **porous bioceramic AV**B. **(C)** under a 100 times magnifying glass; **(D)** under a 1000 times magnifying glass.

The surface characteristics of HA/ZrO_2_ porous bioceramic AVB observated by Ultra-depth microscope showed: the surface of HA/ZrO_2_ porous bioceramic AVBs were coarse, and distributed with irregular three-dimensional interconnected pores inside it (Fig 4). And, the SEM also exhibited that the surfaces were rough and pores distributed non-uniformly (Fig 4).

**Fig 5:**
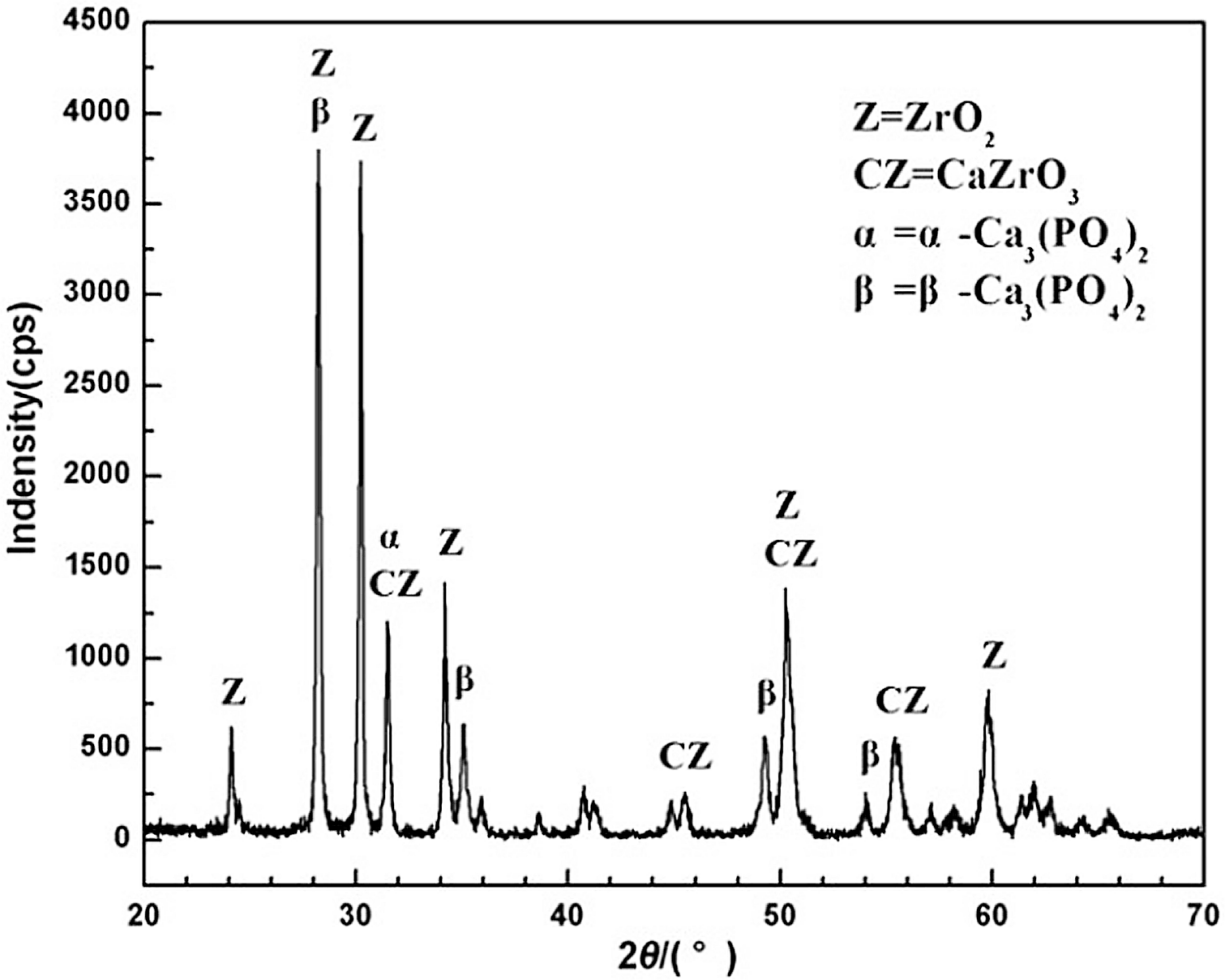
XRD pattern of surface of the HA/ZrO_2_ porous bioceramic.

The X-ray diffraction (XRD) of HA/ZrO_2_ porous bioceramic AVB was shown in Fig 5. After sintering at high temperature, the ZrO_2_ phases of the composite were still remained, but HA phases were disappeared and transformed to β-Ca_3_ (PO_4_) _2_ (β-TCP), α-Ca_3_ (PO_4_) _2_ (α-TCP) and CaZrO_3_ phases. When the HA transformed to β-Ca_3_(PO_4_)_2_ under high temperature, it surely released CaO. However, CaO was not found in the X-ray diffraction of the composite surface, because it was completely dissolved into ZrO_2_, and reacted with it and finally turned into CaZrO_3_ [18]. And after sintering, the occurrences of β-TCP phases were resulted from the enhancement effects of HA’s hexagonal structure. On another side, a small amount of α-Ca_3_ (PO_4_)_2_ phases could also be converted from the β-Ca_3_ (PO_4_)_2_. The above reactions can be expressed as the following reaction formulas:

$$\begin{array}{l} Ca_{10}{\left(PO_{4}\right)}_{6}{\left(OH\right)}_{2}\Leftrightarrow 3\beta -Ca_{3}{\left(PO_{4}\right)}_{2}+CaO+H_{2}O\\ CaO+ZrO_{2}\Leftrightarrow CaZrO_{3}\\ \beta -Ca_{3}{\left(PO_{4}\right)}_{2}\Leftrightarrow \alpha -Ca_{3}{\left(PO_{4}\right)}_{2} \end{array}$$

Furthermore, non-hydrated phosphate phases (TCP phases) treated by the high-temperature, when contacted with water or body fluids, could again be formed as HA at 37°C, and disintegrated Ca^2+^ and HPO_4_^2-^, which would be deposited on the surface of the implanted materials and improved the condition for bone repair. The reaction of HA generated on the surface of TCP was as followed:

$$4Ca_{3}{\left(PO_{4}\right)}_{2}(s)+2H_{2}O\to Ca_{10}{\left(PO_{4}\right)}_{6}{\left(OH\right)}_{2}(surface)+2Ca^{2+}+2HPO_{4}^{2-}$$

### 3.5 General observation

**Fig 6:**
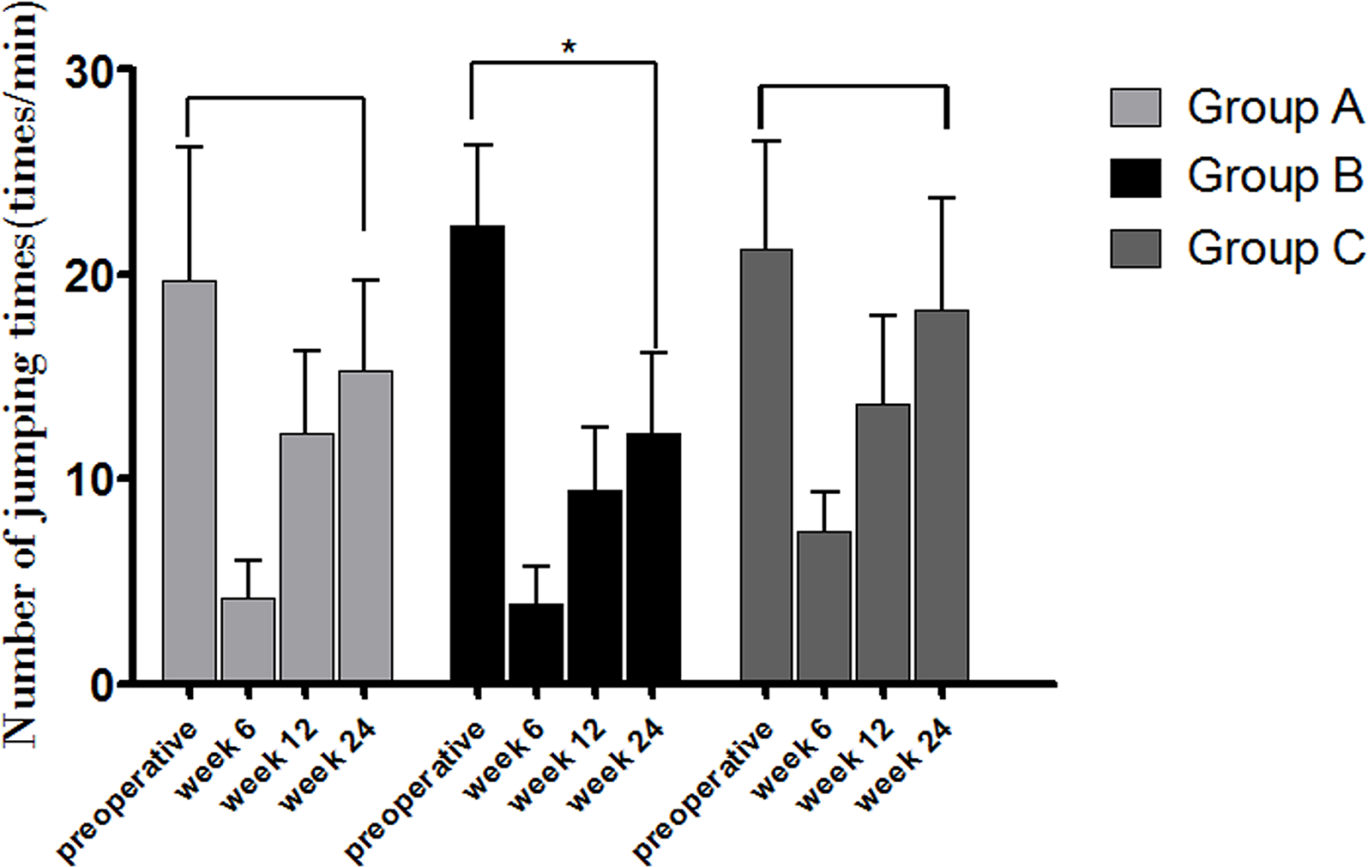
Activities of the beagle dogs during the pre-surgical period and at weeks 6, 12 and 24 after surgery. (* indicates significant difference between the two groups, p<0.05).

Anesthesia recoveries were uneventful for all dogs, and the movements of the limbs were autonomous without complications of incontinency of defecation and urination. There are none the animals became severely ill or died at any time prior to the experimental endpoint. The jump frequencies per min under the same food treats and the same height set were recorded at 6, 12 and 24 weeks after surgery. At 6 weeks postoperatively, animals in group C recovered the fastest with indication of higher average frequencies, compared with groups A and B (p < 0.05); and also at 12 and 24 weeks, when significantly different between group B and group C (p < 0.05) were found (Fig 6). The newly-formed bone within the HA/ZrO_2_ porous bioceramic AVB were found in the retrieved specimens, and the AVBs in groups A and B were fused well with the surrounding pre-existing bone by gross examination at 24 weeks after surgery (Fig 1).

### 3.6 X-ray imaging

**Fig 7:**
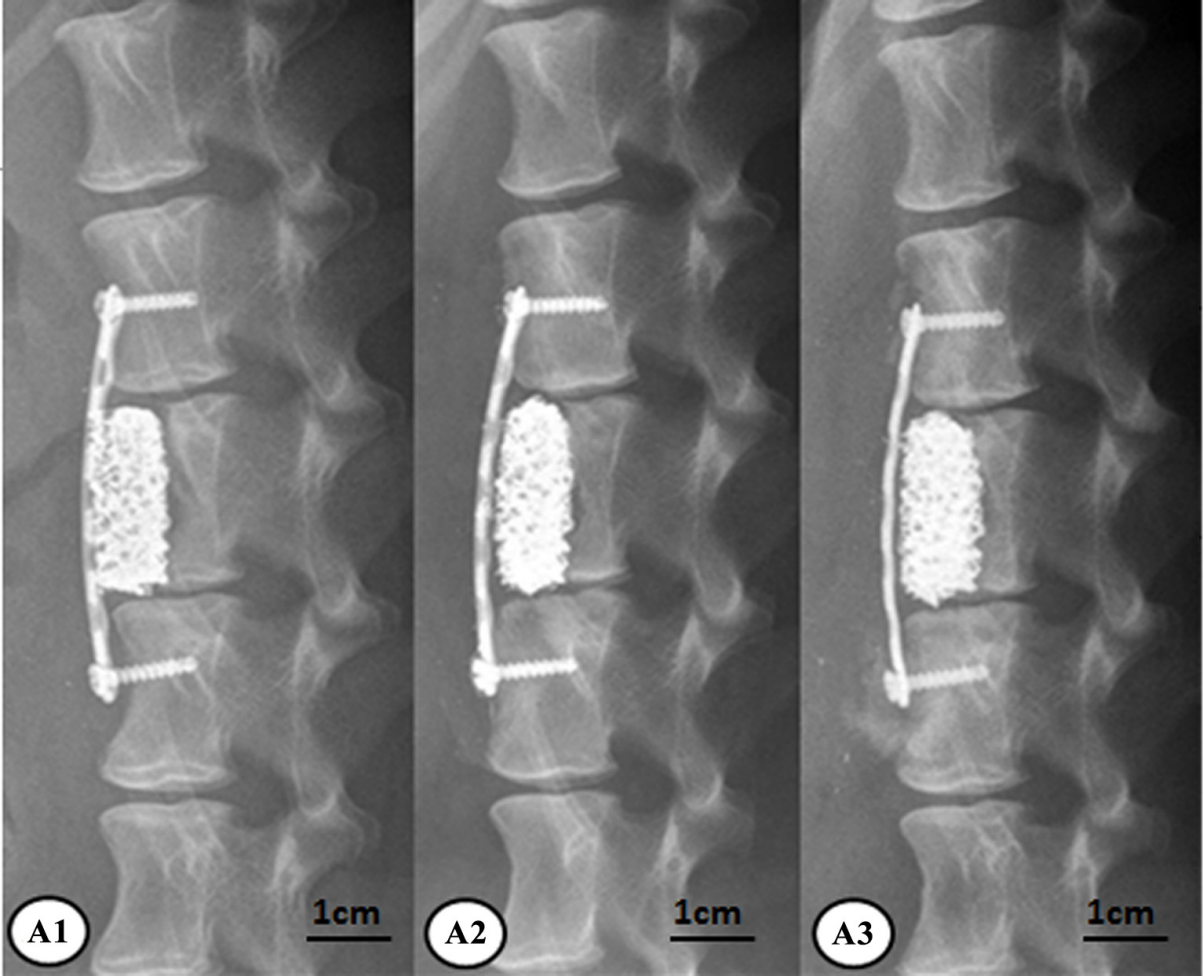
X-ray results of group A after surgery. A1: at week 6, A2: at week 12, A3: at week 24.

**Fig 8:**
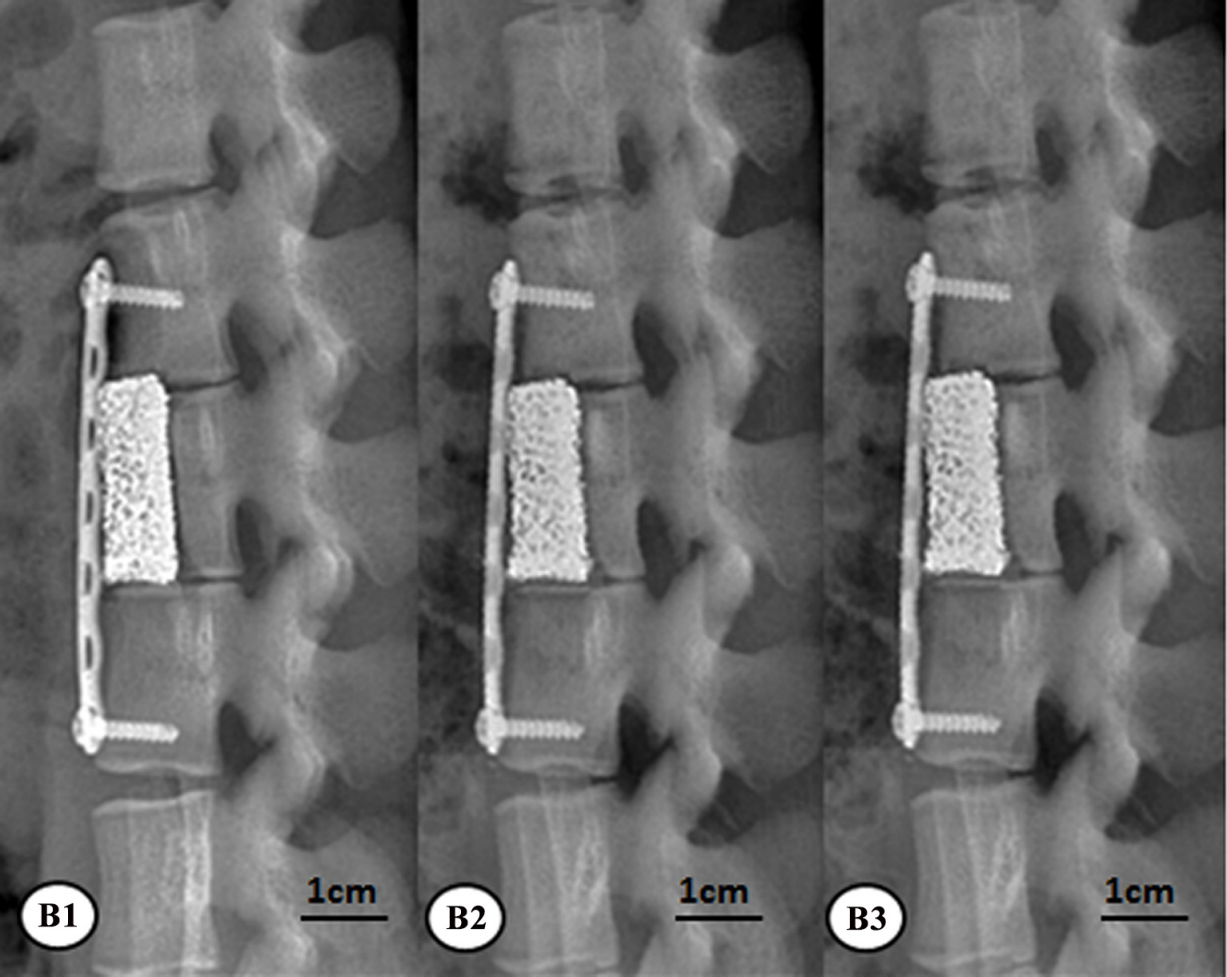
X-ray results of group B after surgery. B1: at week 6, B2: at week 12, B3: at week 24.

**Fig 9:**
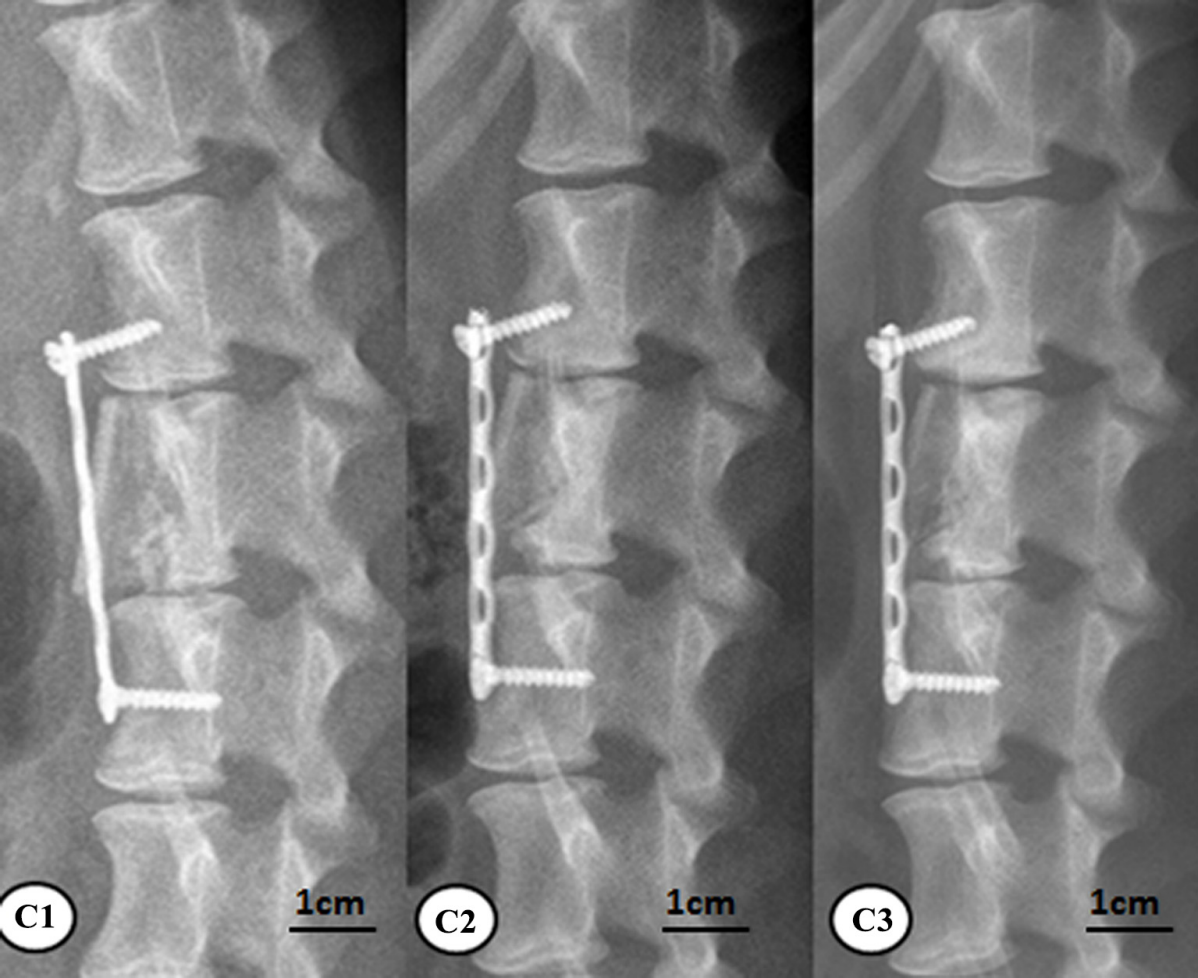
X-ray results of group C after surgery. C1: at week 6, C2: at week 12, C3: at week 24.

At 6, 12, and 24 weeks, animals in all the groups were subjected to the lateral lumbar x-ray imaging under intravenous anesthesia. As to group A, the new bones surrounding the rhBMP-2-loaded/CS AVBs was more prominent at 24 weeks than 6 weeks. The vertebral segments that were subject to the surgery and the corresponding interspinous space were not significantly changed compared with week 6, in addition, the angle of the posterior lumbar was significantly reduced at 24 weeks after surgery (Fig 7). Group B also had prominent new bones surrounding the non-drug-loaded/CS AVB. However, at 24 weeks postoperatively, the angle of the posterior lumbar was not reduced as significantly as those in group A (Fig 8). In group C, the posterior lumbar kyphosis disappeared at 24 weeks with autologous iliac bone grafting, and the autologous iliac bone presented prominent resorption in non-weight-bearing areas (Fig 9). It suggested that the bone remodeling resulted from the autologous iliac bone grafting was slightly faster than groups A and B.

### 3.7 Micro-CT scans and reconstruction of the 3D image

**Fig 10:**
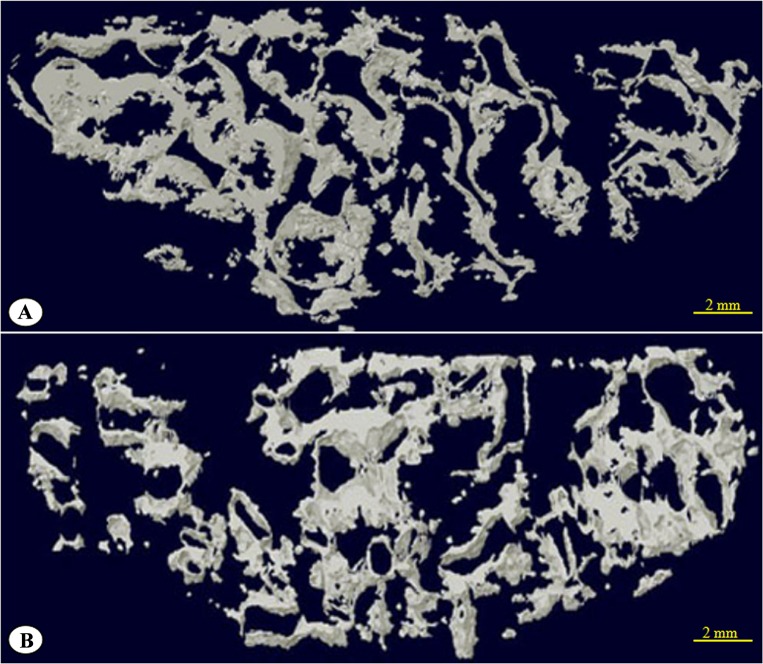
Micro-CT observation of the obtained specimen.

**Fig 11:**
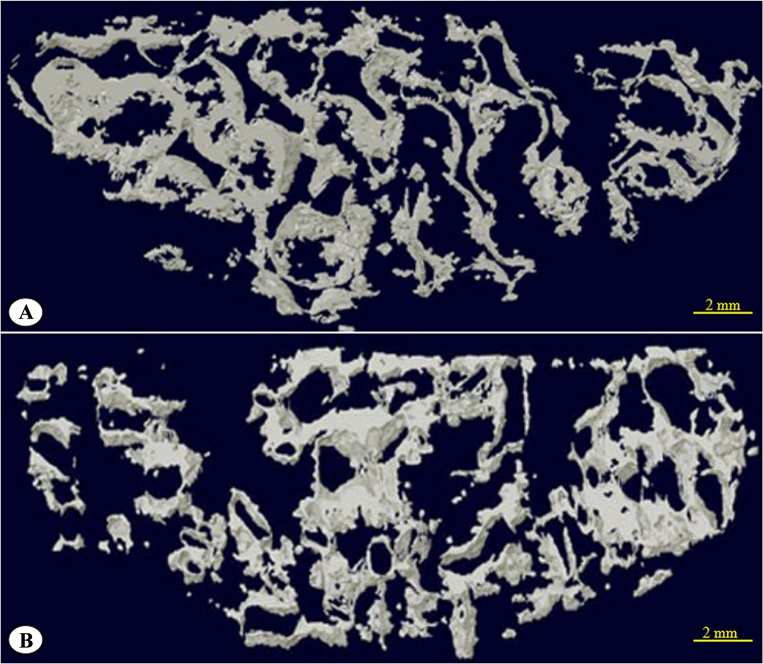
Three-dimensional computed tomography reconstruction of the new bone within 250 μm of the HA/ZrO_2_ porous bioceramic AVB upper surface. (A) group A; (B) group B.

At 24 weeks after surgery, obtained specimens were determined by micro-CT (Skyscan Bruker, Belgium) about the volume of new bone grow into the porous AVB (Fig 10). Micro-CT assessments on the volume of newly-formed bone showed that group A had higher bone content than group B (p < 0.05) (Table 1). The signal selection function of the CT allowed filtering of the ultra-high signals from the material itself and 3-D micro-CT reconstruction of newly-formed bone near the upper surface of the material, within the range of 250 μm, was performed as shown in Fig 11.

**Table 1:** Test results of new bone volume detected by micro-computed tomography at week 24.

Group	bone volume, BV (mm^3^)				(x ± s) mm^3^
A (n = 4)	125.56	150.06	168.76	137.14	145.38±18.52
B (n = 4)	107.65	79.05	71.44	87.07	86.30±15.60

Note: The bone volume within group A material was significantly higher than that within group B (p<0.05)

### 3.8 Biomechanical testing

**Fig 12:**
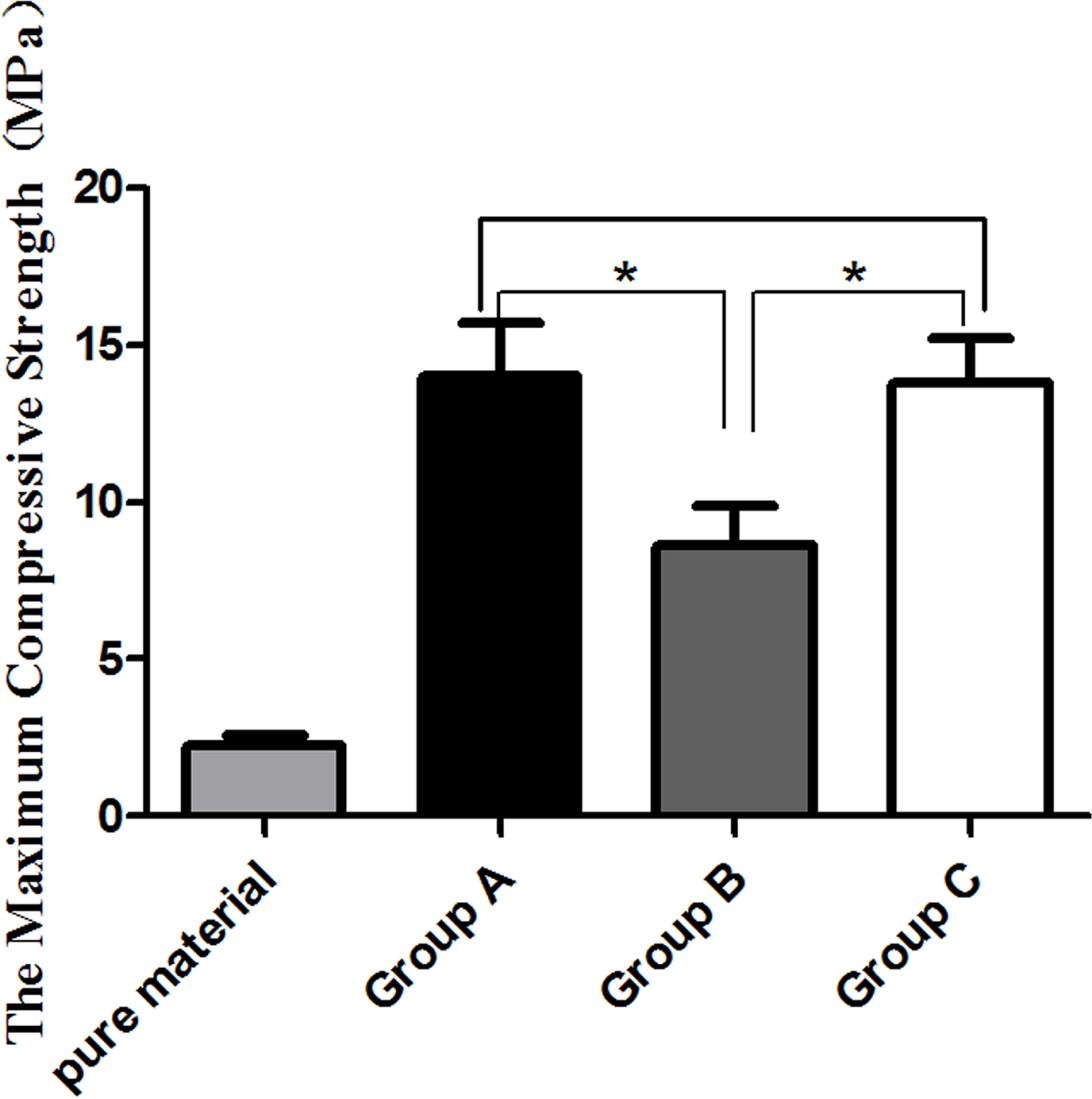
The ultimate compressive strength of the pure AVBs before implantation and in vitro specimens at week 24 after implantation. (* indicates significant difference between two groups, p<0.05).

UCS of the HA/ZrO_2_ porous bioceramic AVB was 2.24 ± 0.36 MPa. At 24 weeks after surgery, the specimens exhibited higher UCS in group A (14.03 ± 1.67 MPa) than group B (8.62 ± 1.24 MPa,) and group C (13.79 ± 1.43 MPa). The UCS of specimens in groups A and C were significantly different from group B (p < 0.05) as shown in Fig 12. The biomechanical test revealed that the UCS of the HA/ZrO_2_ porous bioceramic AVB increased significantly at 24 weeks postoperatively, and the increases in rhBMP-2-loaded/CS AVB was more obvious than the non-drug-loaded/CS AVB in terms of induction of new bone formation and stimulation of the fusion between the grafted material and the autologous bones.

### 3.9 Histological and histomorphometric evaluations

**Fig 13:**
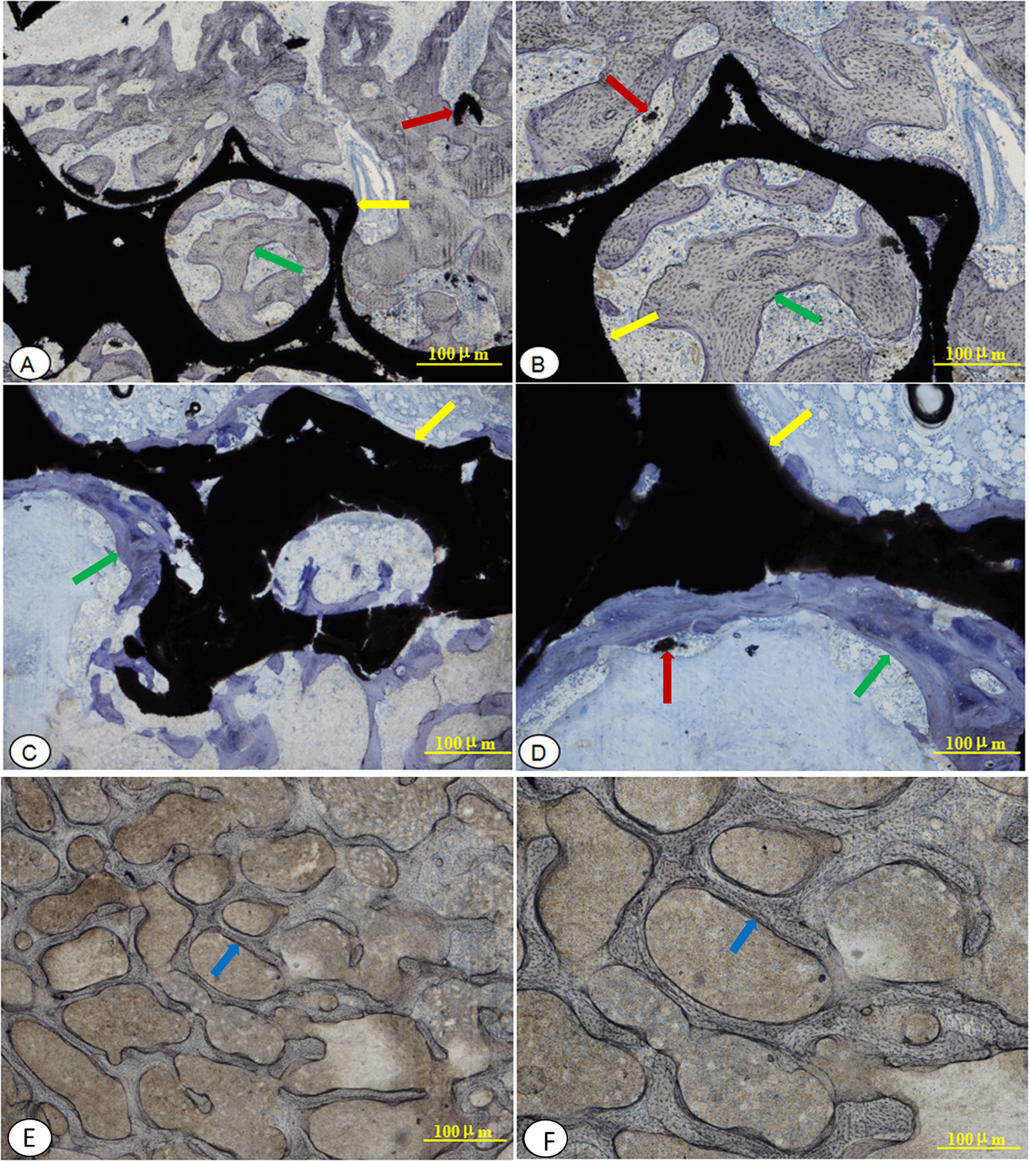
Hard tissue sections stained with toluidine blue light microscopy observations at week 24 after surgery. (A, B): implanted with a rhBMP-2-loaded/CS AVB (group A) A: × 20 magnification; B: × 40 magnification; (C, D): implanted with a non-drug-loaded/CS AVB (group B) C: × 20 magnification; D: × 40 magnification; (E, F):implanted with a autologous iliac bone graft (group C) E: × 20 magnification; F: × 40 magnification; the yellow arrow indicate the implant HA/ZrO_2_-based AVB; the red arrow indicate the HA that under degradation; the green arrow indicate new bone, which was more immature would be tinted deeper blue; and the blue arrow indicate autologous.

Under the light microscopy, the inflammatory cells or body rejection reactions were not found, at the interface between implants and host-bone in each group. At 24 weeks after surgery, material of group A had been integrated tightly into the host bone and filled with a large amount of mineralized bone in pores of the AVB, and new trabecular bone structure were obvious. Toluidine blue staining intensity was lighter and similar to that of the group C, which bone remodeling tended to be well underway (Fig 13). At 24 weeks after surgery, group B displayed a mixture of large number of chondrocytes and osteoblasts at interface between material and host bone, and a lot of osteoid, which exhibited lower bone mineralization degree and intense toluidine blue staining. New bone trabecular structures were scant and more irregular than group A and group C, and the junction between materials and host bone were relatively loose (Fig 13). At 24 weeks postoperatively, group C showed the most mature bone structure situated in the graft area with light toluidine blue staining, and a complete bone fusion with the surrounding pre-existing bone (Fig 13).

## 4. Discussion

The rhBMP-2 has a short biological half-life, degrading too quickly to achieve its expected osteogenic capacity. It is often released in an initial burst rate if uncontrolled, preventing it from attaining the satisfactory biological functions on human bone regeneration [19]. The repair and regeneration of bony defects might take a considerable amount of time, the slower and continuous release of rhBMP-2 is very important especially in a large bony-defect. In contrast, if the times or doses of implanting rhBMP-2 are increased, the side effects and expenses are exacerbated [20–21]. Therefore, to maintain its sustained activity, a number of scholars have attempted to design better systems for the continuous delivery of rhBMP-2 by controlling its release rate [22–24]. Reviewing the literature, the rhBMP-2 was sustainably released from a silica xerogel-chitosan hybrid coating layer on a porous hydroxyapatite scaffold up to 6 weeks [25]. Therefore, CS hybrid material has been used as an ideal carrier for rhBMP-2 to improve its osteogenic induction and to accelerate the repair of large bony-defects. The CS is a natural, non-toxic, biocompatible and biodegradable material. It is the only positively-charged, basic polysaccharide in nature [26]. The positive charge and NH_2_ groups of CS allow for interaction with negatively-charged polymers and macromolecular proteins. The CS used in the present study had good surface morphology, and the manufacturing process was safe and simple to ensure the satisfactory slow-release profile.

In this study, we firstly used STTP as the cross-linker prepared the unloaded, dry CS, and then loaded rhBMP-2 into the CS hydrogel by swelling it in the rhBMP-2 solution. The exposure time of rhBMP-2 to the external environment was reduced significantly and maintained its potency. The cumulative release curve of rhBMP-2 in vitro showed that it was released in stages, with a burst release during the first 3 days, slow release between days 3–12, and steady slow stage from days 12–15. This phenomenon might be related to the following mechanisms: ①the rhBMP-2 uncross-linked to the CS was released by diffusion in the initial burst release; ②the rhBMP-2 covalently linked via special groups was only slowly released as the CS being degraded. After 15 days, the release became steady. However, the released rhBMP-2 content only accounted for 76.79% of the total that was loaded, which indicated a small amount of rhBMP-2 was possibly lost during processing and/or inactivated.

In current work, the novel HA/ZrO_2_-based porous bioceramic AVB with three-dimensional (3D) interconnected spherical pores were prepared by foam immersion, gradient compound and high temperature sintering that were produced by School of Materials Science and Engineering, Shanghai University (Shanghai, China). The physico-chemical characterizations and the biological properties were described previously [27–28]. The porosity of 72.99%–77.48% is set according to the model, which significantly increased the contact area between the HA and the newly-formed bone, and enabled quick fusion after AVB grafting. Additionally, the good biocompatibility, conductivity and osteoinductivity of HA were fully confirmed. Those properties could promote bone fusion and consequently enable HA to be widely applied in basic research and clinical practice as bone substitutes [29]. The partial degradation of HA can release large amounts of Ca^2+^ and HPO_4_^2-^, which would be deposited on the bioceramic surface. The repair of bone defect mainly depends on the induction of new bone formation. This induction process includes both intramembranous ossification and endochondral ossification, and involves the mineralization of new bone, which is a deposition process of calcium phosphate mineral derived from the calcium and phosphate ions in the body fluid. Therefore, to expedite the mineralization of new bone, local concentrations of calcium and phosphate ions need to be exceed the thresholds so to stimulate new bone formation. The increase of the contact area between the HA and the newly- formed bone on the surface can promote the fusion between the grafted material and the host-bones [30]. In contrast, the zirconia (ZrO_2_) has high mechanical properties and low toxicity [31], which is an inert material and cannot be degraded in vivo [32].

In addition, ideal artificial bone materials should have good biocompatibility and allow for quick fusion with the host-bone for excellent mechanical strength. HA/ZrO_2_-based porous bioceramic are biocompatible and do not exhibit cytotoxicity, in vivo acute toxicity, or hemolytic properties [33–34]. In these publications, freshly prepared peripheral blood mononuclear cells (PBMCs) from young adults were cultured with HA/ZrO_2_-based porous bioceramic extracts. Their apoptosis, CD3/CD9 expression, lymphocyte transformation, and cytokine changes were examined to determine the immune-compatibility. Results showed that HA/ZrO_2_-based porous bioceramic exhibited an excellent biocompatibility [35]. The novel HA/ZrO_2_-based porous bioceramic AVB with the 3D interconnection pores in present study showed that dogs with the implantation generally resumed normal activities and appetite. Animals had no general or localized complications during experimental period. Their body temperatures did not increase and immune rejection was not observed. And there was no inflammatory cell or body rejection reaction was found at the interface between implants and host bone on hard tissue sections. All of these suggested that grafting AVBs fabricated from HA/ZrO_2_-based porous bioceramic did not induce an immune reaction from the surrounding host tissues.

According to the 3D CT reconstruction and the observation of hard tissue section at 24 week postoperatively, a large amount of newly formed bone was found within the pores of AVB. Regarding compressive strength of HA/ZrO_2_ composites, researchers reported that the compressive strengths of HA/ZrO_2_ composites, at mixing ratios of 90/10 and 95/5, were approximately at 57 and 48 MPa, respectively [36], and that they, at the ratios of 40/60 and 60/40, were almost at 450 and 300 MPa, respectively [37]. These numerical values are greatly differed from the mechanical strength of the intact lumbar vertebra (1.5–7.8 MPa) [38]. In this study, the HA/ZrO_2_-based porous bioceramic AVB had an in vitro UCS ranging from 2.31–3.1 MPa, which reached 8.26 ± 1.24 MPa at 24 week postoperatively. Meanwhile, the UCS of the AVB complex with the rhBMP-2 CS gel reached 14.03 ± 1.67 MPa at 24 week after surgery, which was similar to that of the grafted vertebral body made from autologous iliac bone. These results suggested the HA/ZrO_2_-based porous bioceramic AVB can repair the spinal defects and show a higher capability of inducing osteogenesis if complex with rhBMP-2 CS gel.

## 5. Conclusions

The current experiment provided useful information as followed. ①The lyophilized CS gel had a three-dimensional (3D) mesh film structure and the size of pores was at between 50–300 μm under SEM. The encapsulation efficiency of rhBMP-2 in CS hydrogel was 91.88 ± 1.53%; and the corresponding loading capacity was 39.84 ± 2.34 ng/mg. ②The rhBMP-2 was released in different stages, such as burst-release for the first 3 days, slower-release in days 3–12, and steady-release between days 12–15. ③The novel HA/ZrO_2_-based porous bioceramic AVB, was prepared by foam immersion, gradient compound and high temperature sintering, with the 3D interconnection pores and porosity of 72.99%–77.48%, which demonstrated excellent biocompatibility and sufficient fracture toughness. ④The novel AVB carried a rhBMP-2-loaded CS gel could induce bone tissue to grow into the pores and promote the repair of bony defect, which provided a secure and robust system as engineered synthetic bone graft substitutes and tissue engineering scaffolds.

## Supporting Information

S1 Fig Agreement of Zhejiang Key Science and Technology project (2014C03031). (TIF)

S2 Fig Agreement of Hanghou Key Science and Technology project (20122513A14). (TIF)

S3 Fig Agreement of Public welfare technology and social development project (2012C33114). (TIF)

S4 Fig Funding statement of the research. (TIF)
