# Supplementary figures and images for: Evaluation of a Novel HA/ZrO2-Based Porous Bioceramic Artificial Vertebral Body Combined with a rhBMP-2/Chitosan Slow-Release Hydrogel
Source: PLoS One. 2016 Jul 11;11(7):e0157698. doi: 10.1371/journal.pone.0157698 (PMC4939960; doi:10.1371/journal.pone.0157698)

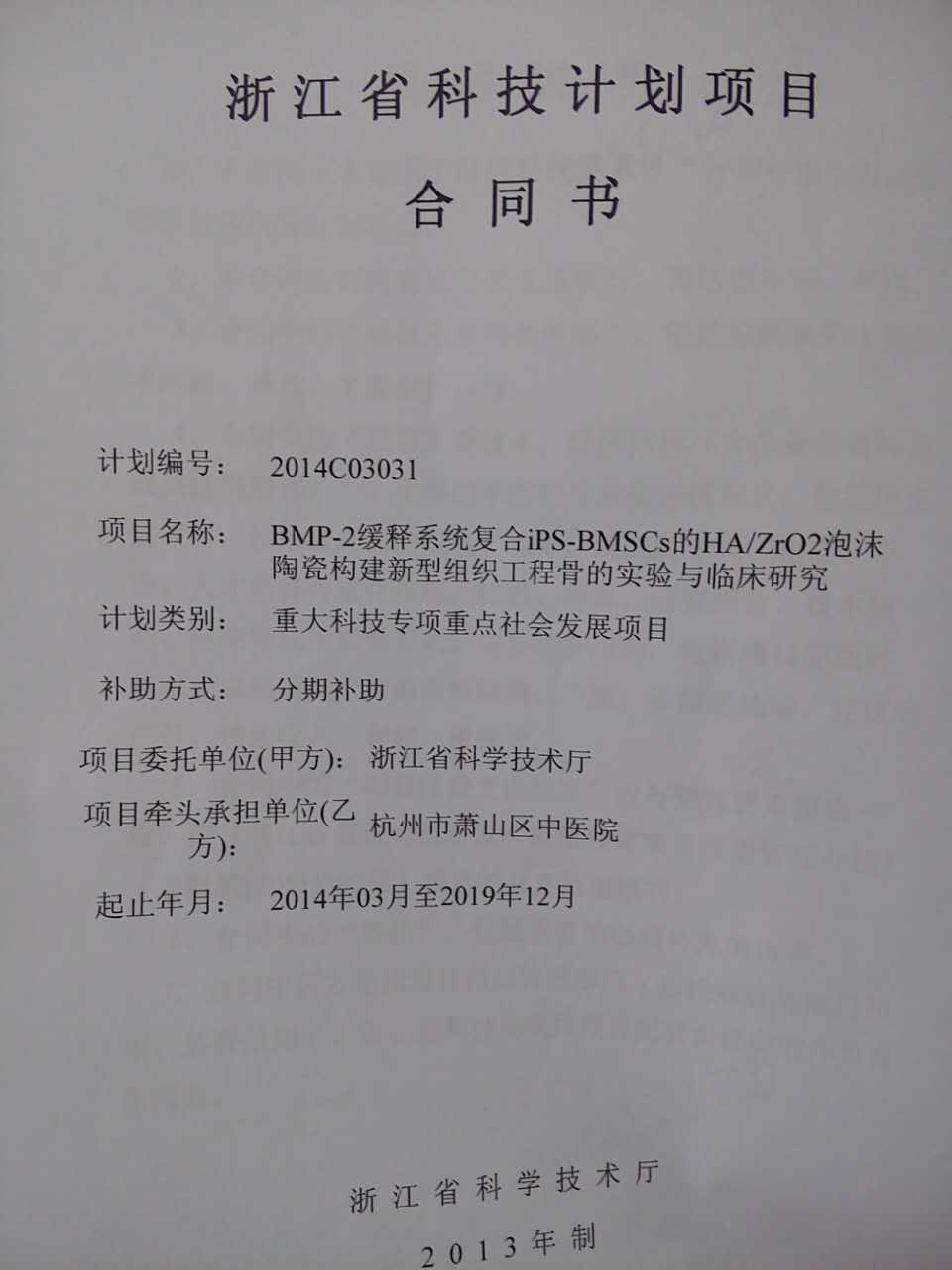

Supplement: S1 Fig — (TIF) [file pone.0157698.s001.tif]

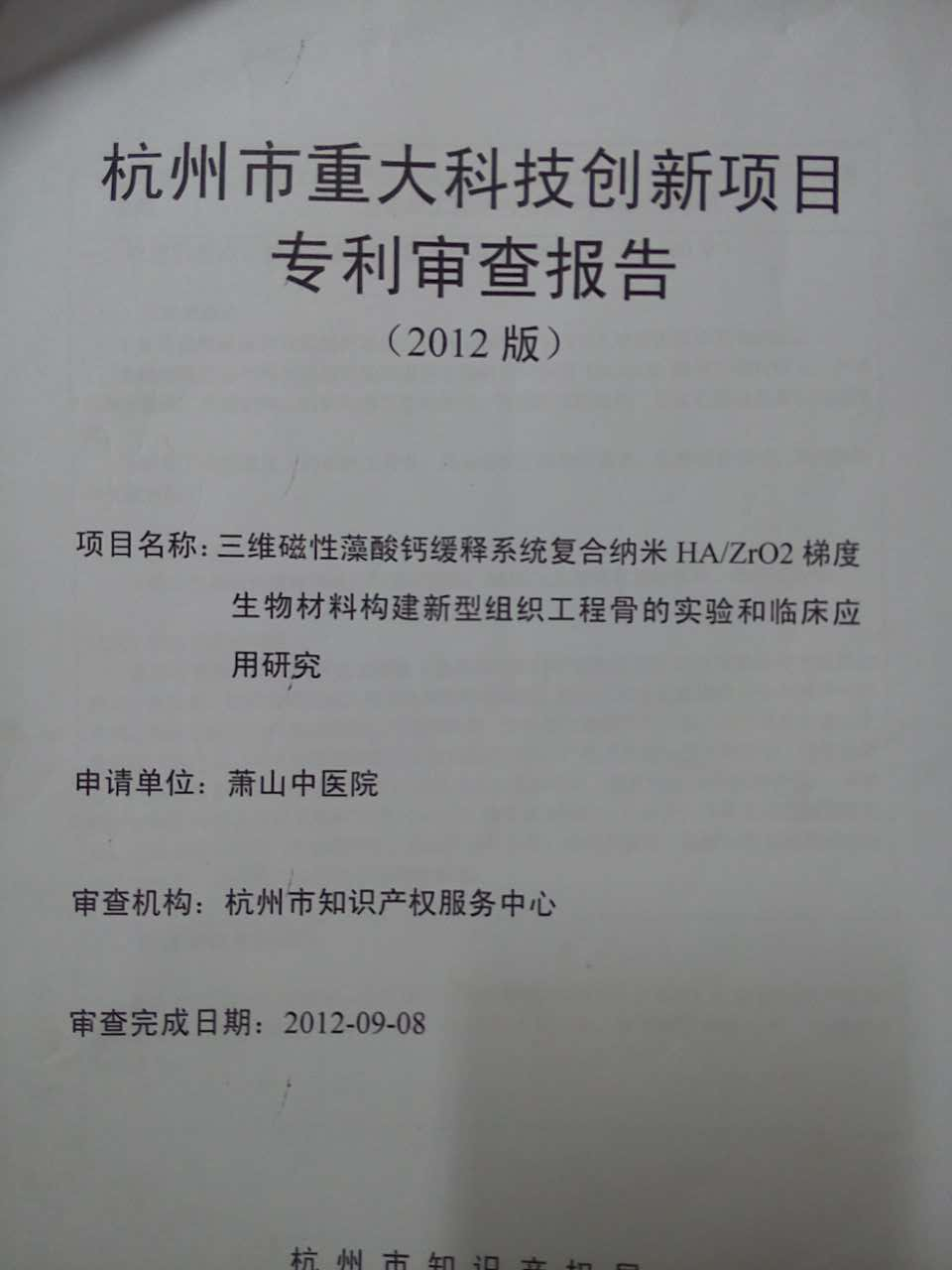

Supplement: S2 Fig — (TIF) [file pone.0157698.s002.tif]

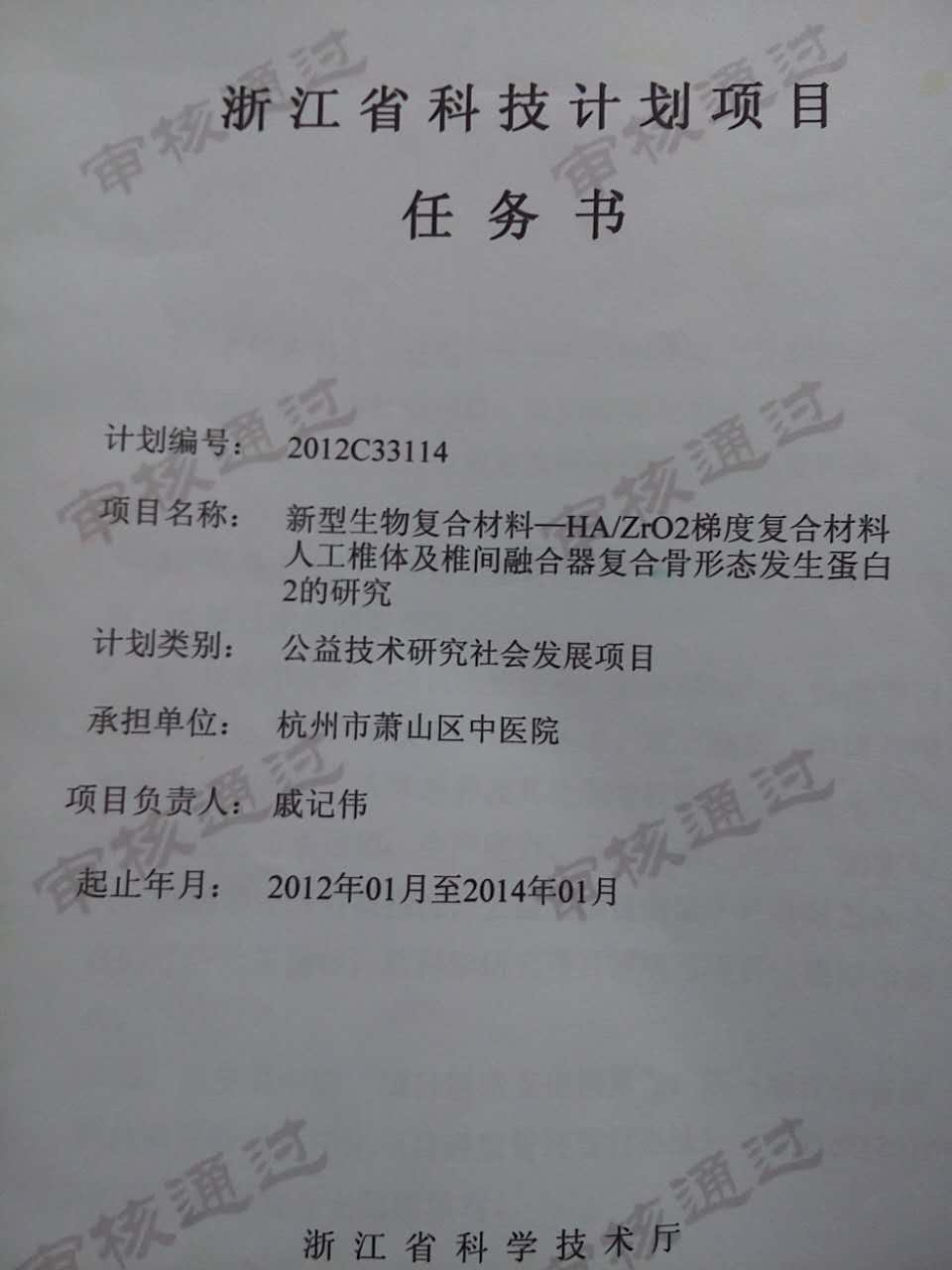

Supplement: S3 Fig — (TIF) [file pone.0157698.s003.tif]

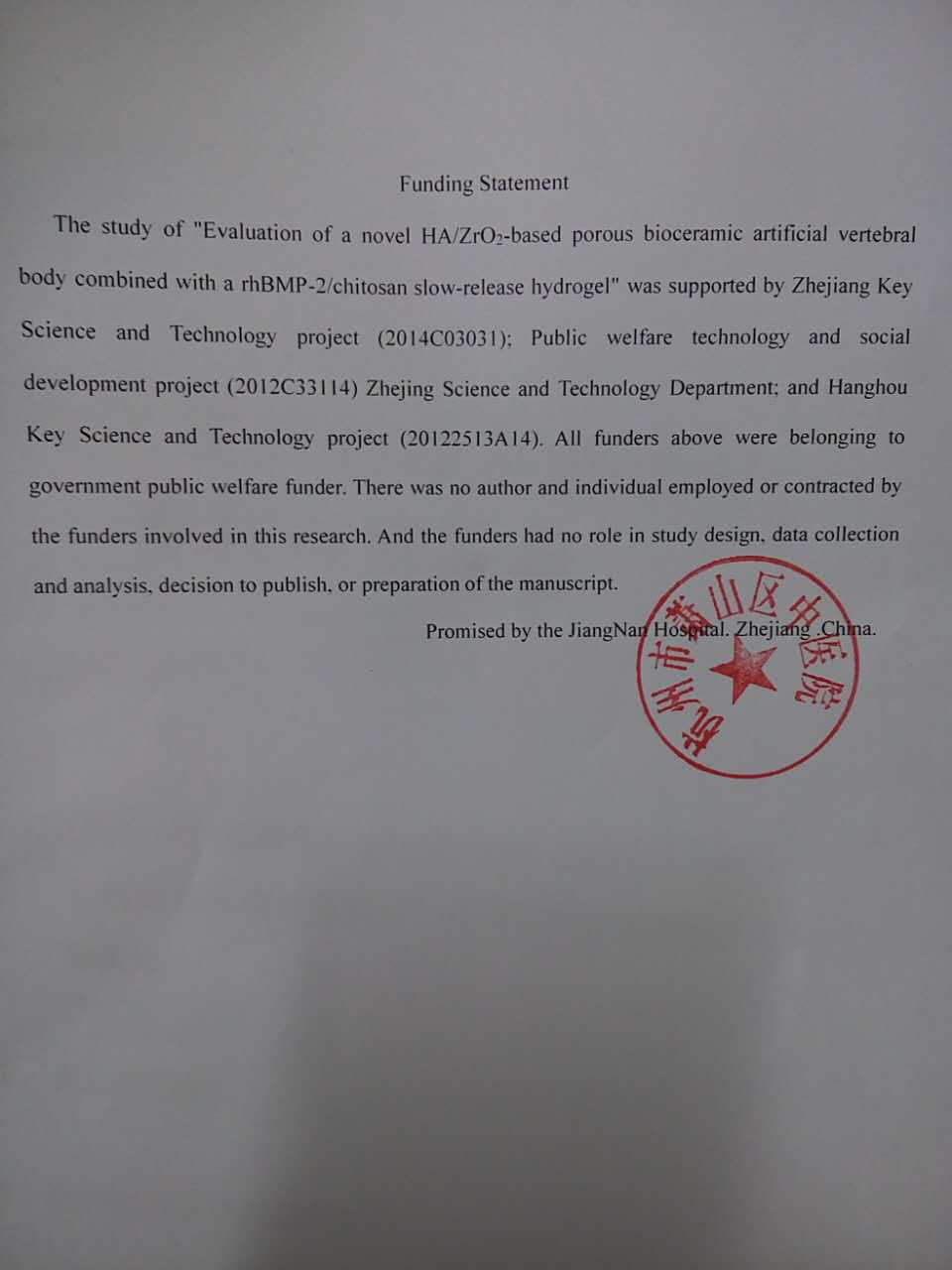

Supplement: S4 Fig — (TIF) [file pone.0157698.s004.tif]
